# Rab4b Is a Small GTPase Involved in the Control of the Glucose Transporter GLUT4 Localization in Adipocyte

**DOI:** 10.1371/journal.pone.0005257

**Published:** 2009-04-17

**Authors:** Vincent Kaddai, Teresa Gonzalez, Frédérique Keslair, Thierry Grémeaux, Stéphanie Bonnafous, Jean Gugenheim, Albert Tran, Philippe Gual, Yannick Le Marchand-Brustel, Mireille Cormont

**Affiliations:** 1 INSERM U895, Centre Méditerranéen de Médecine Moléculaire (C3M), Team 7, Cellular and Molecular Physiopathology of Obesity and Diabetes, Nice, France; 2 Faculty of Medicine, University of Nice/Sophia-Antipolis, Nice, France; 3 INSERM U895, Centre Méditerranéen de Médecine Moléculaire (C3M), Team 8, Hepatic Complications in Obesity, Faculty of Medicine, University of Nice/Sophia-Antipolis, Nice, France; 4 CHU of Nice, Pôle Digestif, Hôpital Archet 2, Nice, France; University of Las Palmas de Gran Canaria, Spain

## Abstract

**Background:**

Endosomal small GTPases of the Rab family, among them Rab4a, play an essential role in the control of the glucose transporter GLUT4 trafficking, which is essential for insulin-mediated glucose uptake. We found that adipocytes also expressed Rab4b and we observed a consistent decrease in the expression of Rab4b mRNA in human and mice adipose tissue in obese diabetic states. These results led us to study this poorly characterized Rab member and its potential role in glucose transport.

**Methodology/Principal Findings:**

We used 3T3-L1 adipocytes to study by imaging approaches the localization of Rab4b and to determine the consequence of its down regulation on glucose uptake and endogenous GLUT4 location. We found that Rab4b was localized in endosomal structures in preadipocytes whereas in adipocytes it was localized in GLUT4 and in VAMP2-positive compartments, and also in endosomal compartments containing the transferrin receptor (TfR). When Rab4b expression was decreased with specific siRNAs by two fold, an extent similar to its decrease in obese diabetic subjects, we observed a small increase (25%) in basal deoxyglucose uptake and a more sustained increase (40%) in presence of submaximal and maximal insulin concentrations. This increase occurred without any change in GLUT4 and GLUT1 expression levels and in the insulin signaling pathways. Concomitantly, GLUT4 but not TfR amounts were increased at the plasma membrane of basal and insulin-stimulated adipocytes. GLUT4 seemed to be targeted towards its non-endosomal sequestration compartment.

**Conclusion/Significance:**

Taken our results together, we conclude that Rab4b is a new important player in the control of GLUT4 trafficking in adipocytes and speculate that difference in its expression in obese diabetic states could act as a compensatory effect to minimize the glucose transport defect in their adipocytes.

## Introduction

Glucose transporter 4 (GLUT4) plays an important role in glucose homeostasis. It is mainly expressed in cells that exhibit insulin-regulated glucose uptake, *i.e* adipocytes, skeletal muscle cells and cardiomyocytes. The intracellular trafficking of GLUT4 is a major determinant of the acute regulation of glucose transport in these cells. In basal state, GLUT4 is present in intracellular locations, referred to as the sequestration compartments, from which it undergoes insulin dependent movement to the plasma membrane [1]. In type 2 diabetic patients, alterations in GLUT4 translocation arise from defects in insulin signalling and in GLUT4 expression [2]. Furthermore, GLUT4 appears mis-located in muscles and adipocytes of diabetic patients [3], [4] or in omental adipocytes of women with gestational diabetes, even when its expression remains normal [5]. Hence, the machinery that regulates GLUT4 trafficking could be altered in insulin-responsive cells of diabetic patients. Understanding the molecular mechanisms controlling GLUT4 trafficking and their alteration in insulin-resistant situations thus represents a major issue for understanding this pathology.

Small GTPases of the Rab family are main organizers of intracellular trafficking [6]. Several Rab members have been involved in GLUT4 trafficking [7], [8], [9]. In particular three Rab proteins of the endosomal recycling system, Rab5, Rab4, and Rab11 participate at different steps of this process [10], [11], [12], [13], [14]. Rab8, Rab10 and Rab14, the putative targets of AS160 [15], a RabGAP inactivated by the insulin-induced PKB pathway [16], were also suspected to play a role in GLUT4 recycling. However Rab8 and Rab14 would act in muscle cells [17] whereas Rab10 would be more important in adipocytes [16]. Rab31, a Rab5 family member, participates in insulin-induced GLUT4 translocation to the plasma membrane. Insulin delocalizes its exchanger Gapex-5 to the plasma membrane and inhibits Rab31 activity, thus potentiating insulin-induced GLUT4 translocation [18].

In the present study, we first aimed at determining whether the expression of Rab proteins of the endocytic recycling pathway was modified in adipose tissues from obese diabetic subjects. We found that Rab4b expression was modified in adipose tissues from both diabetic obese human and mice. It has already been proposed that Rab4a, that possesses 93% of homology with Rab4b, could play a role in GLUT4 trafficking [12], [19], [20], [21], at least in part through its interaction with the Rab4a effector Rabip4 [22] and with the kinesin KIF3B [11]. The role of Rab4b is unknown and we thus aimed at determining whether Rab4b could be involved in the control of GLUT4 localization and glucose transport in adipocytes. First, we showed that Rab4b is mainly located with GLUT4, in its sequestration compartment and in TfR containing endosomes. Rab4b is thus the first Rab protein identified in the GLUT4-sequestration compartment. Second, using different approaches we showed that the inhibition of Rab4b expression resulted in GLUT4 localization changes, with an equilibrium redistribution towards the plasma membranes and probably its sequestration compartments. We thus postulate that Rab4b could be involved in GLUT4 trafficking cycles that allow for GLUT4 intracellular retention in basal conditions.

## Results

### Modifications of some Rabs of the endocytic recycling pathway in diabetes

**Figure 1:**
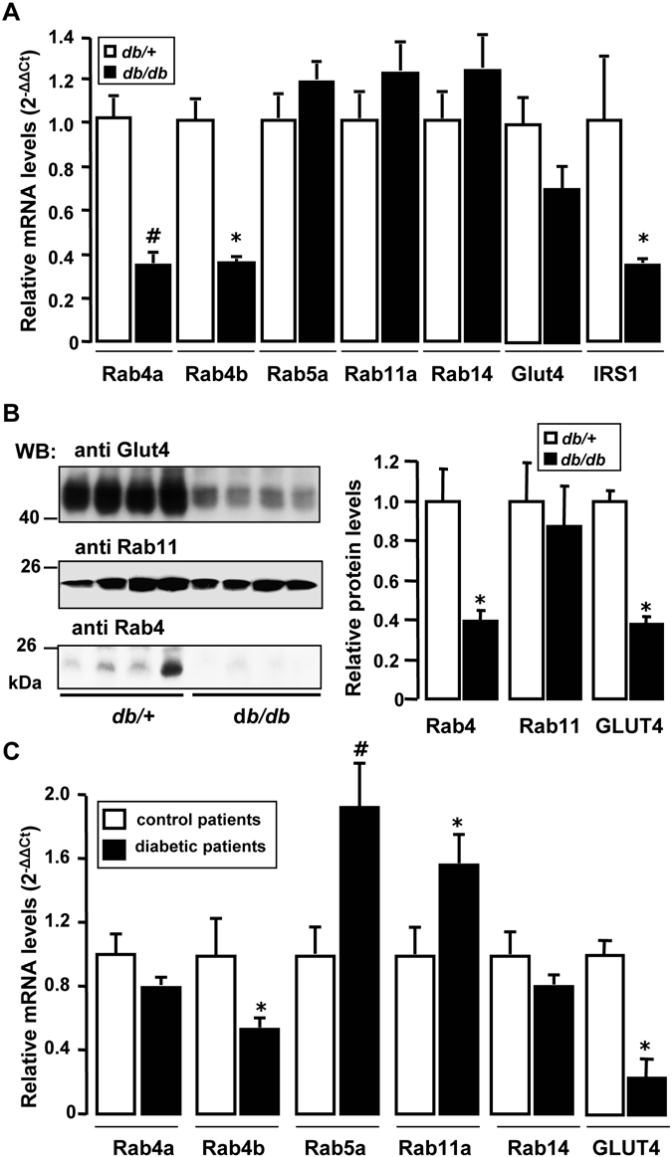
Expression of Rab proteins in adipose tissue of diabetic subjects. A. Total RNAs were prepared from epididymal adipose tissue of 5 *db*/+ and 5 *db/db* mice. The expression levels of mRNA were analyzed by real-time quantitative PCR, normalized to the level of 36B4 mRNA and expressed in arbitrary units with the control values taken as 1. B. Total homogenates were prepared from epididymal adipose tissue of *db*/+ and *db/db* mice. 40 µg of proteins were separated by SDS-PAGE and transferred to PVDF membranes, which were probed with the indicated antibodies. The graph represents the quantification of the amount of Rab4, Rab11 and GLUT4 in 6 *db*/+ and *db/db* mice. C. Total RNAs were prepared from subcutaneous adipose tissue of 5 control subjects and 5 severely obese diabetic patients. The mRNA levels were analyzed by real-time PCR, normalized to the level of RPLP0 mRNA and expressed in arbitrary units as in panel A. Significant with *p<0.05 and ^#^p<0.01 (Kruskal-Wallis test).

Because defects in GLUT4 trafficking could participate in insulin-resistance [3], [4], we determined whether the expression of Rabs involved in endocytic recycling was modified in adipose tissues from obese diabetic mice and humans. Figure 1A shows that the expression of Rab4a and Rab4b mRNAs, and IRS1 mRNA used as a control, was significantly decreased in the epididymal adipose tissues from the obese diabetic *db/db* animals, compared to their lean control (*db*/+) mice. The mRNA expression for Rab5a, Rab11a and Rab14 was unchanged (Figure 1A), as well as those for Rab8a and Rab10 (data not shown). By using an antibody recognizing all Rab4 proteins, we observed a significant decrease in their expression in *db/db* compared to lean *db/+* animals, whereas Rab11 expression was not statistically different (figure 1B). The expression of GLUT4 at the protein level was profoundly decreased in *db/db* mice adipose tissue, although the differences at the mRNA level were not significant. Rab4b mRNA expression was also significantly decreased in subcutaneous adipose tissue from morbidly obese diabetic patients compared to lean subjects (Figure 1C). Rab4a mRNA expression was decreased to a lesser extent, and the difference did not reach statistical significance. In these morbidly obese diabetic patients, the mRNA for Rab5a and Rab11a were increased whereas that of Rab14 was unchanged. The modified expression of the endosomal Rab proteins, especially the inhibition of expression of Rab4b, could thus contribute to the altered functions of adipose tissue in obese diabetic subjects.

### Rab4b mRNA is the predominant Rab4 member in adipocytes

**Figure 2:**
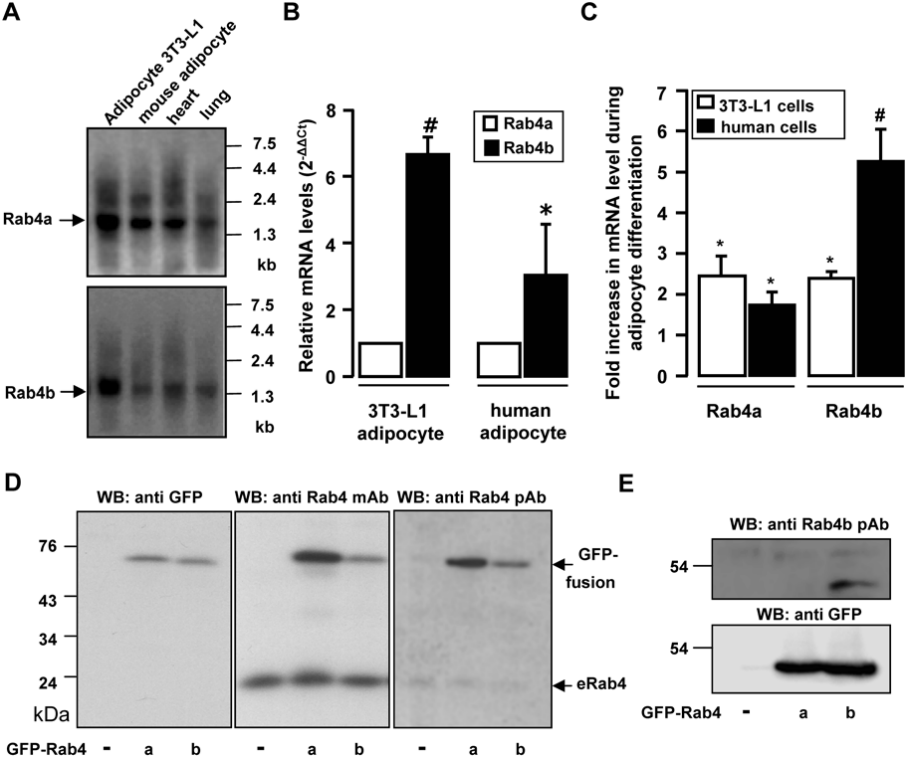
Characterization of Rab4a and Rab4b expression in adipocytes. A. Northern Blot. mRNA from 3T3-L1 adipocytes, isolated mouse adipocytes, heart and lung were analyzed by Northern blot with probes specific for Rab4a or Rab4b. B. Rab4b is the major Rab4 member expressed in adipocytes. RNA was prepared from the indicated cell types. Rab4b and Rab4a gene expression was quantified by real time PCR. The mRNA amounts were calculated by comparison with the control gene expression and the value 1 was given arbitrary to Rab4a mRNA expression. Results were expressed as the mean±SEM of 3 different experiments. Significant differences versus Rab4a expression with p value* <0.05 or ^#^ <0.01. C. Rab4b and Rab4a mRNA expression is increased during adipocyte differentiation. The amount of mRNA for Rab4a and Rab4b was quantified as described in the Materials and Methods section in 3T3-L1 and human preadipocytes and adipocytes. The graph represents the mean±SEM of 3 different experiments of cell differentiation. The results are expressed as the fold increase in adipocytes versus preadipocytes (taken as 1) for the two Rab4 members. Statistical differences between adipocytes and preadipocytes with p value *<0.05 and ^#^<0.01. D. Antibodies produced against Rab4a also recognized Rab4b. Cells were transiently transfected with a mock vector, pEGF-Rab4a, or pEGFP-Rab4b and homogenized. Proteins (40 µg were separated by SDS-PAGE, transferred to PVDF membrane, and probed with anti GFP antibodies, anti Rab4a mAb (BD Biosciences), or anti Rab4a polyclonal [12] antibodies. E. Antibodies produced against Rab4b specifically recognized overexpressed Rab4b. Cells were transiently transfected as in D and proteins were probed with anti GFP antibodies, or polyclonal anti Rab4b antiserum (see Material and Methods).

Because the expression of Rab4 members was modified in total adipose tissues in diabetic state, we further wanted to determine whether these two proteins were expressed in the adipocytes themselves. By Northern Blot analysis, we evidenced the mRNA for Rab4a and Rab4b in freshly isolated and in cultured (3T3-L1) mouse adipocytes (Figure 2A). Rab4a and Rab4b are indeed ubiquitous and we also found their mRNA in lung and heart. By real time PCR, we found that Rab4b mRNA was more abundant than that for Rab4a in 3T3-L1 adipocytes and in human adipocytes differentiated in culture from visceral preadipocytes (Figure 2B). Both Rab4a and Rab4b mRNAs increased during the differentiation of mouse (3T3-L1) and human preadipocytes into adipocytes (Figure 2C). This observation is in accordance with the increased Rab4 proteins we previously reported in 3T3-L1 adipocytes compared to fibroblasts [23]. Thus, Rab4b mRNA is the predominant Rab4 mRNA in adipocytes.

### Expressed Rab4a and Rab4b are co-localized in 3T3-L1 fibroblasts but not in adipocytes

**Figure 3:**
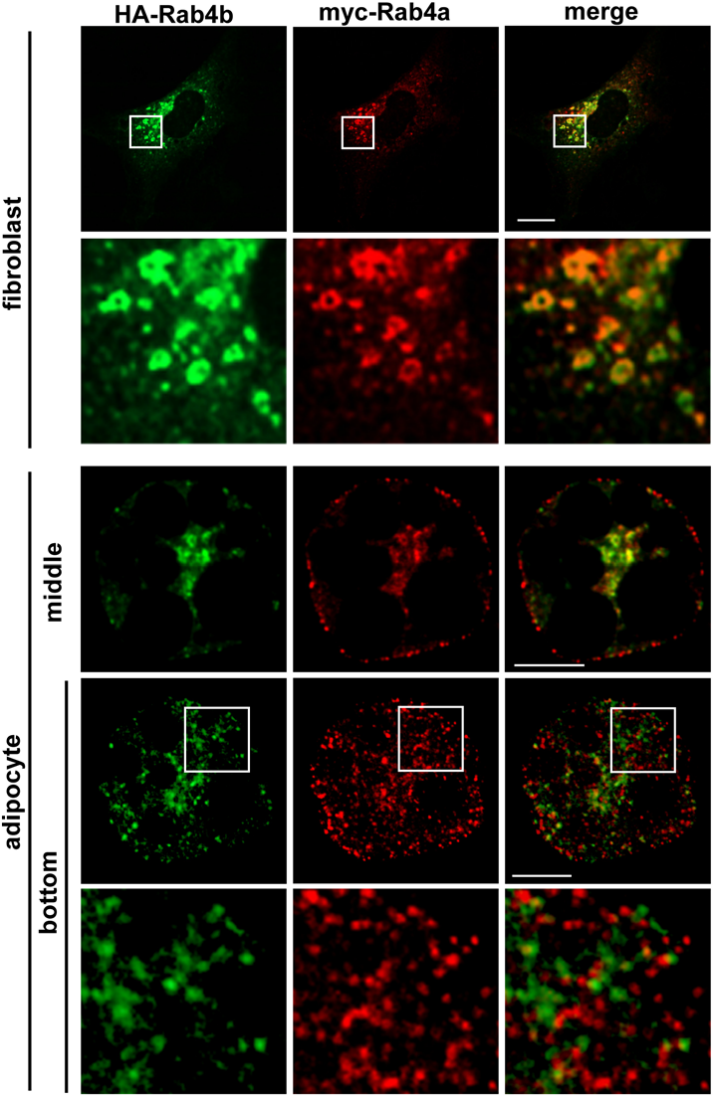
Rab4a and Rab4b are colocalized in 3T3-L1 fibroblasts, but not in adipocytes. 3T3-L1 fibroblasts and adipocytes transiently expressing myc-Rab4a and HA-Rab4b were serum deprived and processed for immunofluorescence. Myc-Rab4a is detected with anti myc mAb and FITC-coupled secondary antibodies. HA-Rab4b is detected with anti HA polyclonal antibodies and TexasRed-coupled secondary antibodies. A confocal section of the double-transfected cell, and the corresponding enlarged views of the delineated areas are shown. Each labeling (green and red) as well as the merge images are shown. Cells with comparable levels of fluorescence were chosen. Bar is 1 µm.

We next wanted to compare Rab4b and Rab4a localization in adipocyte in order to determine whether they potentially could fulfill different functions. The characterization of the two Rab4s at the protein level requires specific antibodies. Because available monoclonal and polyclonal antibodies despite they were supposed to be specific for Rab4a also recognized Rab4b in Western Blot (Figure 2D), we developed antibodies against the C terminal specific part of Rab4b. The obtained antiserum recognized overexpressed Rab4b but not Rab4a (Figure 2E), but unfortunately, none of those antibodies detected endogenous levels of Rab4 proteins in indirect immunofluorescence. We thus compared the localization of expressed Rab4b and Rab4a fused to different tags in 3T3-L1 cells. Hemagglutinin (HA)-Rab4b and myc-Rab4a were mostly present and colocalized in the perinuclear region of 3T3-L1 fibroblasts (Figure 3). Interestingly, when expressed in adipocytes, HA-Rab4b and myc-Rab4a were mainly associated with different vesicles dispersed into the cytoplasm, whereas a partial colocalization was detected in the perinuclear region (Figure 3). This observation suggests that Rab4b, when adipocyte differentiation occurs, is partly segregated from the early endosomes, which are labeled by Rab4a in many cell types [24], [25].

### Rab4b is largely colocalized with GLUT4 or VAMP2 and to a lower extent with the transferrin receptor in 3T3-L1 adipocytes

**Figure 4:**
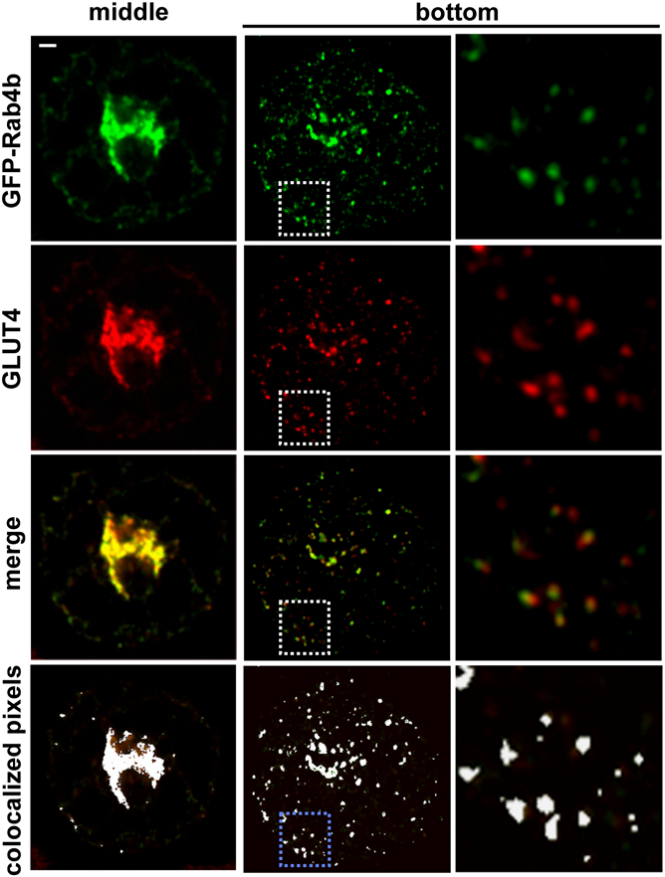
Rab4b is associated to the GLUT4 containing compartments. Adipocytes stably expressing GFP-Rab4b were serum deprived overnight before being processed for immunofluorescence. GFP-Rab4b is detected using anti GFP mAb followed by FITC- coupled secondary antibodies whereas GLUT4 is detected by goat anti GLUT4 and TexasRed-coupled secondary antibodies. Two confocal sections of the same cell, obtained in the middle and at the bottom, are shown for the two fluorochromes or merge by using Adobe Photoshop software. Enlarged views of the delineated areas are in the right column. Colocalization of the two proteins results in a yellow color. Colocalization was also visualized by using the ImageJ colocalization finder pluging. Pixels over a fixed threshold where a green and red fluorescence were depicted with a ratio 1/1 are shown in white on the merge image. Bar is 1 µm.

**Figure 5 pone-0005257-g005:**
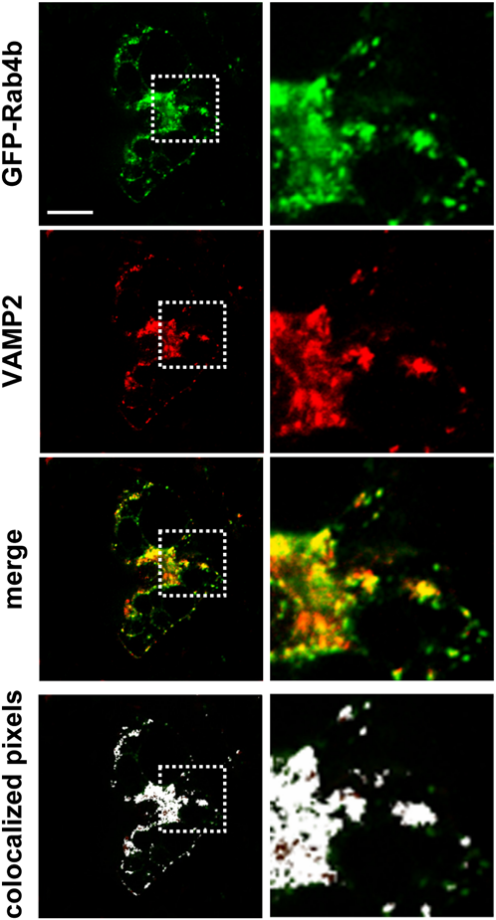
Rab4b is present in the VAMP2-containing compartment. In cells obtained as described in Figure 4, GFP-Rab4b is detected by anti GFP rabbit polyclonal antibodies and FITC-coupled secondary antibodies. VAMP2 is detected by mAb followed by TexasRed-coupled secondary antibodies. One middle confocal section is shown. Enlarged views of the square delineated with continuous lines are shown in the right panels. Colocalisation was also visualized by using the ImageJ colocalization finder pluging. Bar is 1 µm.

**Figure 6:**
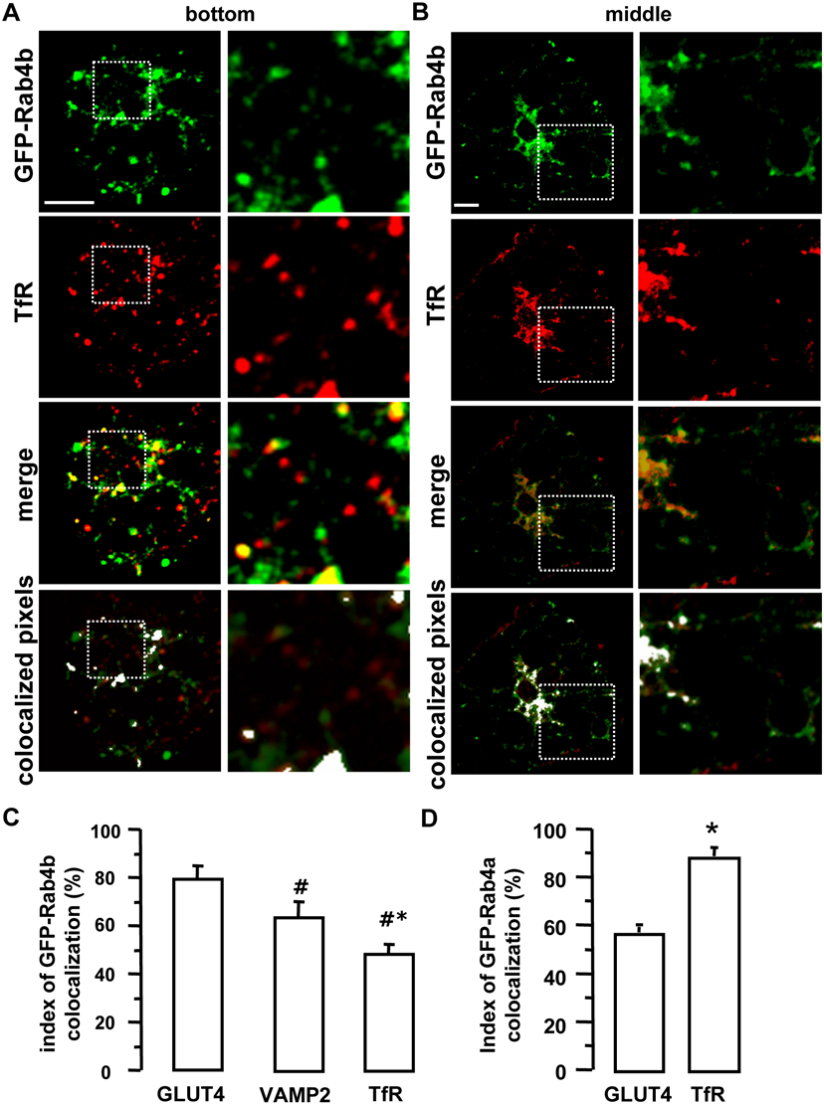
GFP-Rab4b is partially colocalized with TfR. GFP-Rab4b expressing cells were obtained as in figure 4. TfR is detected by mAb followed by TexasRed-coupled secondary antibodies. Confocal sections obtained in the bottom (A) and one in the middle (B) of an adipocyte are shown. Enlarged views of the delineated areas are in the right columns. Each labeling (green and red) as well as the merge images are shown. Bar is 1 µm. Colocalization was also visualized by using the ImageJ colocalization finder pluging. C. The quantification of the colocalization between GFP-Rab4b and the indicated proteins (corresponding to figures 4–6) was performed by using the image correlator plus function of ImageJ. The index of colocalization corresponds to the mean of the overlap coefficient (R)*100 obtained for more than 20 cells for each co-labeling. The ratio between green and red signals is comprised between 0.8 and 1.2. *indicates a significant difference in the index of Rab4b colocalization with VAMP2 compared with GLUT4, whereas ^#^ indicates a significant difference in the index of Rab4b colocalization with TfR compared with VAMP2, the two p being <0.01 (Kruskal-Wallis test).

We next aimed at determining the Rab4b compartment in adipocyte. We established cell lines overexpressing green fluorescent protein (GFP)-Rab4b at a low level, where we verified that the fusion protein GFP-Rab4b behave similarly than HA-Rab4b (data not shown). This low expression level of GFP-Rab4b (less than 2 fold, supplementary Figure S1) did not allow for the direct detection of the GFP by fluorescence microscopy but requires the use of anti-GFP antibodies in all the following experiments. We first compared the subcellular distribution of GFP-Rab4b with GLUT4 and with VAMP2 (vesicle-associated membrane protein 2), a marker of the GLUT4 sequestration compartment [1]. In 3T3-L1 adipocytes, GFP-Rab4b was enriched in a perinuclear region and associated with small vesicles throughout the cytoplasm (Figure 4). GFP-Rab4b was highly co-localized with endogenous GLUT4 in those two compartments, with an index of 80%. On a similar fashion, most of GFP-Rab4b was present in structures that contain endogenous VAMP2 (Figure 5) and around 65% of the two proteins were colocalized (Figure 6C). By contrast, the extent of colocalization between GFP-Rab4b and the transferrin receptor (TfR), a marker of early sorting/recycling endosomes did not overcome 50% (Figure 6A–C), which is similar to the colocalization extent described between GLUT4 and TfR [1]. In another adipocyte cell line that overexpressed GFP-Rab4b by nearly 6 fold, we also observed that the extent of colocalization of Rab4b is higher with GLUT4 than with Tfr (Figure S1). In adipocytes that transiently co-expressed GFP-Rab4b and GLUT4, the two proteins were also mainly co-localized whereas it is not the case with GFP-Rab4a (Figure S1). These observations indicate that Rab4b is present in the compartments where GLUT4 is mainly localized in basal condition *i.e* its sequestration compartment and also in TfR containing endosomes. In accordance with the low colocalization of Rab4a with Rab4b in adipocytes, we did find a higher colocalization index between Rab4a and Tfr than between Rab4a and GLUT4 (Figure 6D, and supplementary Figures S2, S3).

### Effects of the down-regulation of Rab4b on glucose uptake

**Figure 7:**
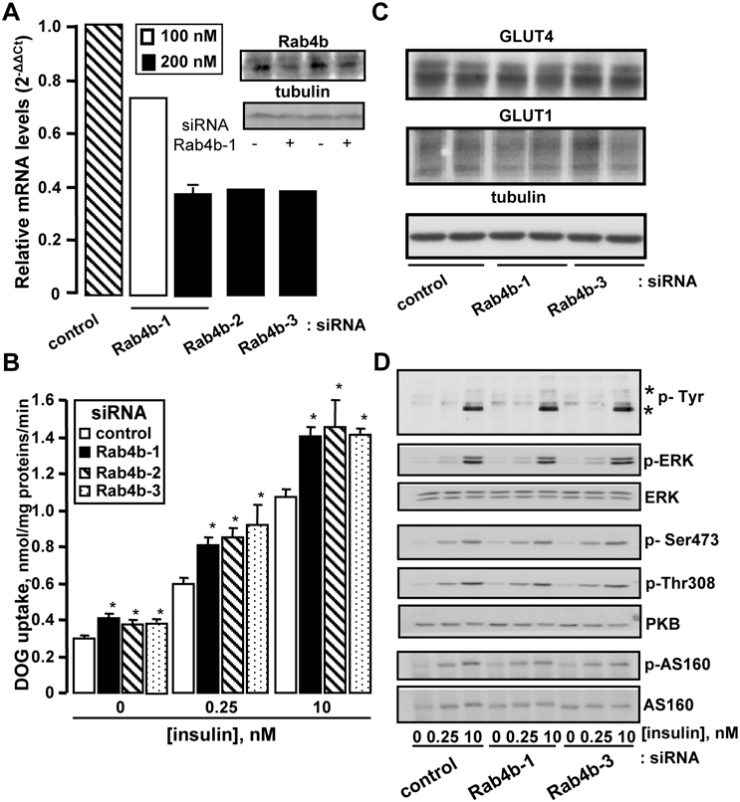
Effect of the downregulation of Rab4b on deoxyglucose uptake in adipocytes. A. 3T3-L1 adipocytes were transiently transfected with control siRNA or the three siRNA against Rab4b at the indicated concentration. 48 h later, the amount of Rab4b mRNA was quantified by using real-time PCR. Graph represents the relative mRNA levels for Rab4b in cells treated with Rab4b siRNA compared with cells treated with the corresponding concentration of control siRNA. No differences in Rab4b mRNA expression were observed with the two concentrations of control siRNA, or with cells treated without siRNA or with INTERFERin™ alone. In the inset is shown the immunodetection of Rab4b, and tubulin as a loading control, of adipocyte homogenates treated with control or anti Rab4b-1 siRNA and prepared 72h after the transfection. B. Cells were transiently transfected with 200 nM of control siRNA or of the three siRNA against Rab4b. 72 h later, cells were serum deprived for 2 h, then treated or not with the indicated concentrations of insulin, before measurement of deoxyglucose uptake, as described in Methods. The figure represents the mean±SEM of 4–6 experiments. * indicated results that are statistically significant compared with control siRNA-treated cells using paired student t test (p<0.005). C. Adipocytes were transfected as in A with 200 nM of the indicated siRNA and total homogenates were prepared 72 h later. 40 µg of proteins were resolved by SDS-PAGE. GLUT4, GLUT1, and tubulin as a loading control were then detected by immunodetection. D. 3T3-L1 adipocytes were transfected with the indicated siRNA. 72 h later, they were serum deprived for 2 h, then treated without or with the indicated concentrations of insulin for 10 min. Total homogenates were prepared and 40 µg of protein were resolved by SDS-PAGE. Tyrosine phosphorylated proteins, phosphorylated PKB, AS160, total PKB and tubulin were revealed by using specific antibodies. Asterisks indicates phosphorylated insulin receptor and its substrates IRS.

To determine the effect of Rab4b on glucose uptake, we performed its siRNA-mediated knockdown in 3T3-L1 adipocytes. Figure 7A shows that 200 nM of the siRNA against Rab4b-1 was required to inhibit by 60% the expression of Rab4b mRNA. Inhibition of the Rab4b protein expression was revealed by using the antiserum we have produced against the C terminal part of Rab4b (Insert Figure 7A) and its extent reached 53.4%±1.2 (n = 5). The siRNA Rab4b-1 did not affect the expression of Rab4a mRNA (see Figure S4). Two other siRNA against Rab4b directed against different Rab4b regions (Table S1) were also able to inhibit Rab4b expression (Figure 7A) but not Rab4a mRNA expression (data not shown). The decreased expression of Rab4b through the use of these three different siRNA caused an increase in the ability of adipocyte to transport DOG in basal (25%) and insulin conditions (40%), either at low or maximally stimulating concentrations (Figure 7B). The effect of insulin is thus not affected by the down regulation of Rab4b expression. The down-regulation of Rab4a up to 80% by using two different siRNA did not induce significant changes in basal or in insulin-induced glucose uptake (Figure S4). The down regulation of Rab4b expression induced this increase in glucose uptake without modifying the expression of GLUT4 and GLUT1 (figure 7C), the glucose transporters responsive for glucose transport in 3T3-L1 adipocyte [26]. It did not affect either insulin-stimulated phosphorylation of the insulin receptors, IRS, PKB, AS160, or ERK (Figure 7D and Figure S5 for quantifications).

### Effects of the down-regulation of Rab4b on GLUT4 localization

**Figure 8:**
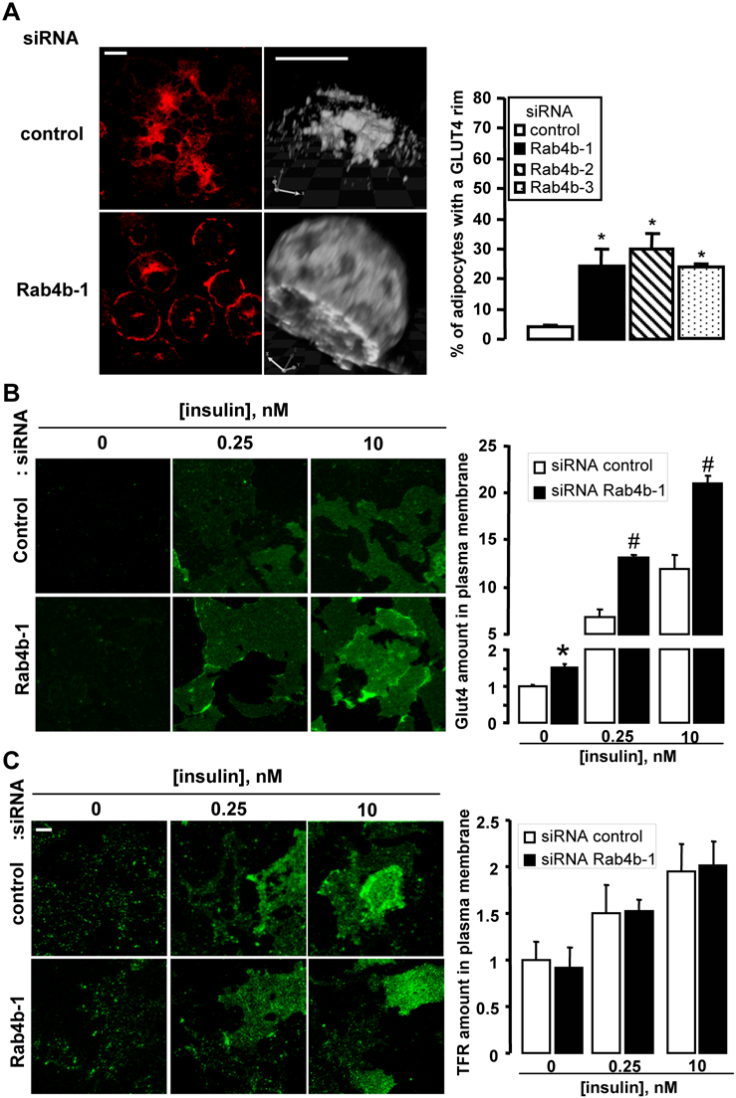
A decreased expression of Rab4b increased GLUT4 localization at the plasma membrane. A. Cells were transiently transfected with 200 nM of control siRNA or of the indicated siRNA against Rab4b. 72 h later, cells were serum deprived for 2 h and processed for endogenous GLUT4 immunodetection. Typical fields and a 3D reconstruction of one adipocyte performed with the Volocity software are shown as well as the quantification of the number of adipocytes with peripheral labeling (rim) of GLUT4 (bar 10 µm). About 100 cells were examined in at least 10 different fields for each condition. The experiment was reproduced 3 fold and the figure shows the mean±SEM of these experiments. * indicates that the differences were significant with p<0.001 using the Kruskal-Wallis test relative to control siRNA-treated cells. B.C. Adipocytes were transiently transfected with 200 nM of control or anti Rab4b-1 siRNA. 72 h later, cells were serum deprived, treated with the indicated concentration of insulin, and used to prepare plasma membrane lawns. GLUT4 and Tfr were detected by indirect immunofluorescence in B and C, respectively. Representative fields were shown and the quantification of the amount of GLUT4 or TfR was performed as indicated in the Material and Methods section. The differences between control and siRNA treated cells are significant with p<0.01* and p<0.001 (Kruskal-Wallis test).

**Figure 9:**
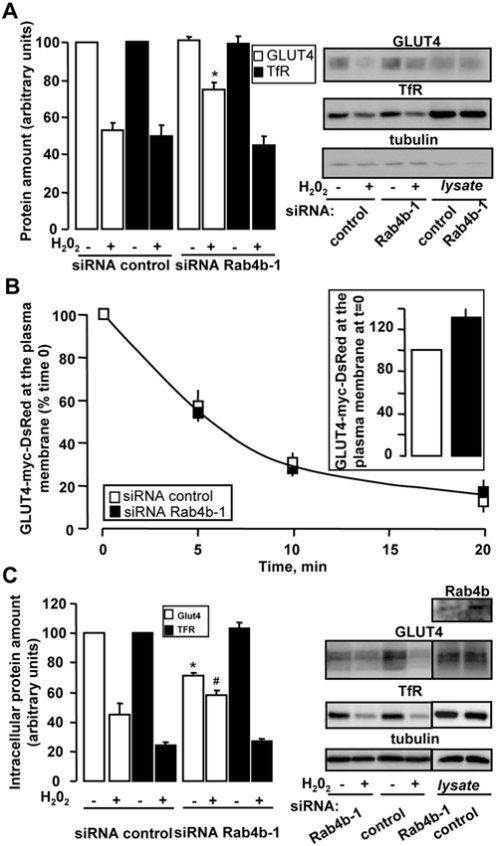
Effect of a decreased Rab4b expression on GLUT4 location by using Tf-HRP-induced endosomal ablation. A. 3T3-L1 adipocytes were transiently transfected with 200 nM of control or anti Rab4b-1 siRNA. 72 h later, cells were serum deprived and treated as indicated in the Material and Methods section for the ablation of endosomes. Total homogenates were prepared from 3T3-L1 adipocytes loaded with Tf-HRP for 2 h at 37°C without or with performing the ablation of the endosomes (minus and plus H_2_O_2_, respectively). Representative immunoblots for GLUT4, TfR, and tubulin as a loading control not affected by the endosomes ablation, were shown, as well as the quantification of 3 independent experiments. An aliquot of total lysate from cells treated with control or anti Rab4b-1 siRNA was also analyzed to determine the effect of Rab4b downregulation on the expression level of GLUT4 and TfR. B. 3T3-L1 adipocytes stably expressing myc tagged-GLUT4-DsRed were transiently transfected with 200 nM of control or anti Rab4b-1 siRNA. 72 h later they were treated for an internalization assay as described in the Materials and Methods section. The inset shows the amount of GLUT4-myc-DsRed inserted within the plasma membrane following insulin stimulation. The graph represents the GLUT4-myc-DsRed at the plasma membrane at various periods of time following the induction of internalization processes expressed as the % of the amount detected at time 0. The results are expressed as the mean±SE of two independent experiments. C. Adipocytes were treated as in A and intracellular vesicles were prepared as described in the Material and Methods section. Representative immunoblots for GLUT4, TfR, and tubulin as a loading control, were shown, as well as the quantification of 3 independent experiments. An aliquot of total lysate from cells treated with control or anti Rab4b-1 siRNA was also analyzed to determine the effect of Rab4b downregulation on the expression level of GLUT4 and TfR.

We thus aimed at determining whether a redistribution of GLUT4 at the plasma membrane could explain the stimulatory effect of Rab4b down regulation on glucose uptake. By using three experimental approaches we found that the down regulation of Rab4b expression was accompanied by a change in GLUT4 subcellular location towards the plasma membrane. First, we observed in basal conditions an increased number of adipocytes with a GLUT4 rim, a characteristic image of adipocyte with plasma membrane GLUT4 (Figure 8A). Interestingly, the down regulation of Rab4a did not induce obvious GLUT4 subcellular distribution modifications (Figure S4). Second, the GLUT4 immunoreactivity increased in plasma membrane lawns prepared from basal and insulin-stimulated adipocytes (Figure 8B), without any concomitant modification of the plasma membrane content of TfR (Figure 8C). Third, using a technique to render insoluble (ablated) intracellular compartments containing the TfR by means of a transferrin-horse radish peroxidase conjugate (Tf-HRP) [27], we observed that the efficiency of endosomal ablation is modified for GLUT4 but not for TfR. Endosomal ablation (condition with H_2_O_2_) resulted in a 50% decrease in the amount of GLUT4 in whole adipocyte homogenates of control cells, compared with a 25% reduction in anti Rab4b siRNA treated cells (Figure 9A), thus confirming that GLUT4 localization in basal condition was modified when Rab4b expression is decreased. Furthermore, we observed that the binding of anti myc antibodies on intact insulin-treated adipocytes increased following Rab4b down-regulation in an adipocyte cell line expressing a myc tagged GLUT4-DsRed, a glucose transporter with an extracellular myc eptitope (Figure 9B). The increase in glucose transport following insulin treatment observed in anti Rab4b siRNA-treated adipocytes could thus also be explained by an increase in GLUT4 at the plasma membrane.

The increase in the amount of GLUT4 at the plasma membrane could result either from an increase in its exocytosis or an inhibition of its endocytosis. We thus determined whether the down regulation of Rab4b could modify GLUT4 internalization (Figure 9B). We observed that internalization of extracellularly myc tagged-GLUT4-DsRed was similar in cells transfected with control or anti Rab4b siRNA, thus strongly suggesting that inhibition of Rab4b expression favours GLUT4 exocytosis.

In whole cell homogenates, proteins insensitive to endosomal ablation could be located at the plasma membrane and/or in a non endosomal intracellular compartment (see [28]). Further, an increase in GLUT4 amount at the plasma membrane in absence and in presence of insulin could result from a redistribution of GLUT4 from its sequestration compartments toward the endosomes [29]. We thus wanted to determine whether such GLUT4 redistribution could explain the effect of Rab4b downregulation on basal and insulin-stimulated glucose transport. We thus needed to estimate the amount of GLUT4 in endosomal and non-endosomal compartments. For that, adipocytes treated or not with anti Rab4b siRNA were processed for Tf-induced endosomal ablation and enriched-intracellular vesicles were prepared and analyzed for TfR and GLUT4 content (Figure 9C). In agreement with previous reports [27], [28], 80% of intracellular TfR containing vesicles were ablated (condition with H_2_O_2_) whatever Rab4b was present or down-regulated. The results differed totally for GLUT4. First, Rab4b down regulation decreased the intracellular amount of GLUT4 (condition without H_2_O_2_), which confirms our previous series of observations i.e. an increase of GLUT4 at the plasma membrane. Second, very few of these vesicles were sensitive to ablation (condition with H_2_O_2_). Because the total GLUT4 amount was unchanged in lysates, the results suggest that in normal condition nearly half of intracellular GLUT4 were outside the TfR containing endosomes and, more importantly, that GLUT4 appears to be mainly excluded from endosomes when Rab4b is down regulated.

## Discussion

Rab proteins are main players of intracellular vesicular traffic because they control the transport of vesicles between two adjacent organelles [6]. The identification of Rab proteins associated with the GLUT4 sequestration compartments represents a major challenge to understand the GLUT4 recycling pathway. Several Rabs including Rab2A, Rab4, Rab8A, Rab10, Rab11, and Rab14 have been identified as proteins of immunopurified GLUT4-containing structures [7], [12], [15], [30], [31], [32] using methods that do not discriminate between the various GLUT4-containing intracellular organelles. It is thus not surprising that a large number of Rabs has been found associated with GLUT4, because GLUT4 traffics through numerous organelles on top of its sequestration compartment [1].

The method of choice to determine which Rabs localize in GLUT4 sequestration compartment is thus indirect immufluorescence using confocal microscopy. The lack of antibodies recognizing endogenous Rab in immunofluorescence has likely restrained such studies, and only one describes a small colocalization of Rab14 with GLUT4 positive vesicles [32]. The Rab proteins specifically associated with the GLUT4-sequestration compartments thus remain to be identified and our results strongly suggest that Rab4b is one of them. Indeed, we found Rab4b widely located with GLUT4, thus indicating that under basal condition Rab4b localized in most of the numerous GLUT4 compartments, including the GLUT4-sequestration one. In accordance, the majority of VAMP2-containing structures, known to correspond to the GLUT4 sequestration compartments, are also labelled by Rab4b. Rab4b is thus the first Rab of these specialized compartments, but it is also found in TfR-containing endosomes. This endosomal localization in adipocytes could explain why Rab4b is mainly colocalized with the endosomal marker Rab4a in fibroblasts and not in adipocytes. Indeed, the GLUT4 sequestration compartment is absent in fibroblast and is formed along the adipocyte differentiation process [33]. Our data concerning Rab4b localization are strengthened by the fact that the extent of colocalisation between Rab4a and GLUT4 is low, whereas Rab4a is essentially colocalized with TfR. To circumvent the lack of antibodies able to detect endogenous Rab4b by immunofluorescence, we used the GFP-tag as the Rab location reporter. This fusion protein is predicted to have similar functions and thus the same location as the wild type Rab4b. Indeed, N-terminal GFP fusion with Rab proteins does not affect their functionality [34] and GFP-Rab4b co-localized with the Rab4b fused with a smaller tag (HA-Rab4b). We used a retrovirus infection in order to obtain a low level of exogenous Rab4b expression unlikely to impair their localization and organelle morphology (less than 2 fold). We further observed that a higher expression of GFP-Rab4b did not impair its extent of colocalization with GLUT4.

Because Rab4b is present in early endosomes as well as in the GLUT4 sequestration compartment, it could play a role in its traffic between these two compartments. If such is the case, the inhibition of Rab4b function should interfere with GLUT4 recycling and probably with insulin-induced glucose transport. Decreasing the expression levels of Rab4b mRNA by 60% yields to a small increase in basal glucose uptake and a more sustained effect at a physiological and maximal concentration of insulin. The insulin effect remained similar in control and anti Rab4b siRNA treated adipocytes. The down regulation of Rab4a expression, by contrast, did not changed basal nor insulin-induced glucose transport. However, previous studies using overexpression of Rab4a [12] or of the Rab4a effector Rabip4 led to the conclusion that Rab4a was involved in GLUT4 trafficking [22]. One explanation could be that Rab4b could fulfil Rab4a function in its absence. It should also be kept in mind that Rab4a is weakly expressed compared to Rab4b in adipocyte. Further, Rabip4 is an effector of both Rab4a and Rab4b [35].

**Figure 10:**
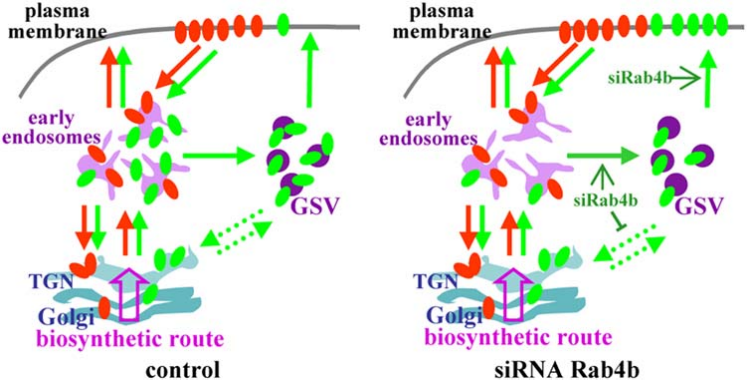
Schematic representation of the putative role of Rab4b in GLUT4 trafficking control. TfR (transferrin receptor in orange) and GLUT4 (in green) continuously recycle between intracellular compartments and the plasma membrane. However their routes are divergent. TfR only used the early endosomes (sorting and recycling ones) as intracellular reservoir whereas GLUT4 is also found in the *trans* Golgi network and in the GLUT4 sequestration vesicles (GSV). Because down regulation of Rab4b affects GLUT4 location, but not that of TfR, it is suspected to play a role in GLUT4 specific trafficking step(s). Its role would be to retain GLUT4 intracellularly by controlling positively or negatively one or more steps of trafficking involved in these retention loops.

This increase in glucose uptake observed with Rab4b siRNA treatment could be explained by the presence of more GLUT4 molecules at the plasma membrane, as evidenced by multiple experimental approaches. Our results indicate that decreasing the amount of Rab4b perturbs the location of GLUT4, but not that of TfR. Rab4b would thus act on a trafficking step which does not involve the classical endosomal recycling, but specifically concerns the GLUT4-sequestration compartment. Furthermore, the increase in plasma membrane GLUT4 in basal condition does not appear to result from a decrease in GLUT4 endocytosis or from a transfer of GLUT4 towards the endocytic compartments, as indicated by the Tf-HRP induced endosomal ablation. This would be indeed predicted from recent results showing that an increase in GLUT4 amount at the plasma membrane in basal and insulin conditions could be due to a redistribution of GLUT4 from its sequestration compartment to the endosomes [29]. Thus, a possible mechanism could be that Rab4b is involved in the intracellular retention machinery of GLUT4 both in basal and in insulin-stimulated conditions. Rab4b could be involved in a GLUT4 cycle between the sequestration compartment and plasma membrane or/and between the sequestration compartment and other intracellular organelles, as schematized in figure 10. The function of Rab4b seems however to differ from that of Rab31. Indeed, Rab31 knock-down does not favour plasma membrane GLUT4 localization in basal condition. Furthermore, Rab31 potentiates insulin action [18], whereas the efficiency of insulin to recruit GLUT4 at the plasma membrane is unaffected by the inhibition of Rab4b expression. Rab4b function also differs from that of Rab4a. Indeed, in absence of coupling of Rab4a with its effector Rabip4, GLUT4 traffic between endosomes and the sequestration compartment is altered [22]. When Rab4b expression is decreased, intracellular GLUT4 is mainly in its sequestration compartment thus suggesting that trafficking between endosomes and the sequestration compartment occurs. The next steps will be to determine the exact trafficking event(s) controlled by Rab4b. The challenge will be to develop new experimental strategies to invalidate more efficiently Rab4b and to characterize specific Rab4b effectors in adipocytes.

A consistent down-regulation of Rab4b mRNA expression in adipose tissues of severely obese and diabetic patients and animals was observed. According to our *in vitro* results, this decrease could thus contribute to modify GLUT4 trafficking in adipocytes. However, these observations are paradoxical because a decreased Rab4b expression positively regulates insulin-induced glucose transport in cell systems whereas insulin-induced glucose transport is altered in adipose tissue from obese subjects. Although not testable, this decrease could contribute to fight adipocyte insulin resistance by increasing resting glucose uptake in order to compensate the decreased adipocyte GLUT4 expression, a common feature of many insulin resistant states [36] that we also observed in our partients. Presently, there is no proof that the proportion of plasma membrane GLUT4 is increased in insulin resistant situations although it has been suggested in two studies [4], [37]. Indeed, it was shown that the plasma membrane content of IRAP, which recycles like GLUT4 in adipocytes and has an unaltered expression, is increased in plasma membranes of adipocytes isolated from diabetic patients [4]. Changes in intracellular GLUT4 and/or IRAP location between control and diabetic patients were also evidenced in adipocyte and muscle by using fractionation procedures [3], [4], [5]. Further studies will be needed to characterize the subcellular distribution of GLUT4 and IRAP in insulin resistant situations. Altogether, our results documents Rab4b as the first Rab protein which co-localize in adipocytes with GLUT4 in its sequestration compartments which is modulated in diabetes.

## Materials and Methods

### Antibodies

Goat polyclonal antibodies against GLUT 4 and anti myc epitope mAb (9E10) were from Santa Cruz Biotechnology (Santa Cruz, CA, USA). Anti VAMP2 and GFP mAb was from Synaptic Systems (Göttingen, Germany). Rabbit polyclonal antibodies against GFP were from BD Biosciences Clontech (Palo Alto, CA, USA). Anti Rab4 mAb was from BD Biosciences Pharmingen (San Diego, CA, USA). Anti transferrin receptor (TfR) monoclonal antibodies (mAb) and anti Rab11 polyclonal antibodies were from Zymed Laboratories Inc (San Fransisco, CA, USA). Rabbit polyclonal against GLUT1 was from Abcam (Cambridge, UK). Antibodies against Phospho-PKB (Thr308) and PKB were from Cell Signalling (Beverly, MA, USA). Antibodies against Phospho-AS160 were from Biosource (Camarillo, CA, USA). Anti tubulin mAb was from Sigma (Saint Louis, MO, USA). Rabbit polyclonal antibodies against Rab4a were produced as described in [12]. Horseradish peroxidase-conjugated and fluorochrome conjugated secondary anti species antibodies were from JacksonImmunoResearch Laboratories, Inc (West Grov, PA, USA). TrueBlotTM ULTRA HRP anti mouse IgG was from eBioscience (San Diego, CA, USA). Antibodies against Rab4b were obtained by immunizing rabbits with a 12 amino acids peptide corresponding to the C terminal part of Rab4b coupled to KLH (Eurogentec, Seraing, Belgium).

### Reagents

DNA modifying enzymes were from NEW ENGLAND Biolabs, Inc (Beverly, MA, USA), chemicals from Sigma (St. Louis, MO, USA). SYBR green were Eurogentec (Seraing, Belgium), kits for DNA purification from Qiagen (Valencia, CA), cell culture media from Invitrogen (Carlsbad, CA). Oligonucleotides were synthetized by Eurogentec (Seraing, Belgium) and their sequences are available upon request. We used the large P0 subunit of the acidic ribosomal phosphoprotein-RPLP0 as an invariant control [38], [39]. We also used TaqMan primers for human Rab4a and Rab4b (Applied Biosystem). A siRNA against mouse Rab4b was designed and synthetized by Eurogentec (siRNA-Rab4b-1, Seraing, Belgium). siRNA-Rab4b-2 is from Dharmacon. siRNA-Rab4b-3 is from Ambion (Austin TX, USA). Their sequences were provided in supplementary Table S1. Control siRNA was from Eurogentec. HRP-conjugated mouse transferrin was from JacksonImmunoResearch Laboratories, Inc (West Grov, PA, USA).

### cDNAs and Plasmids

Mouse Rab4b cDNA is the RIKEN clone AK005314. The coding sequence of Rab4b was amplified by PCR and subcloned into pEGFP-C1 (BD Biosciences Clontech) to obtain a N-terminal GFP fusion protein. The sequence encoding GFP-Rab4b, GFP-Rab4a [40], and GLUT4-myc-DsRed [22] were subcloned into the Snab1 site of pBabe^puro^, allowing for the production of retrovirus used to infect 3T3-L1 fibroblasts. The amplified Rab4b coding sequence was sub-cloned in pcDNA3-HA (Invitrogen, Carlsbad, CA) to obtain N-terminal HA tagged Rab4b. Other Rab4a constructions used were described in [41].

### Animals

Fed *db*/+ and *db/db* male mice (C57BL/KSJ-*mLep^db^*/+ or *mLep^db^*/*mLep^db^*), obtained from Janvier (Le Genest St Isle, France) were studied at 13 weeks of age. Fed glycemia were measured using a blood glucose meter and were 31.6±0.72 and 10.0±0.3 mM in *db/db* and *db*/+ mice, respectively (mean±SEM of 5 mice). Animal experimentation was conducted in agreement with accepted standards of animal care. The experiment protocol has been reviewed and accepted by our Institutional Ethic Animal Care Committee.

### Human subcutaneous adipose tissue biopsies

Five morbidly obese diabetic women (age 49±9 years, BMI 44±7 kg/m^2^, fasted glycemia 11.7±3 mM, HbA1c, 9±2%) were recruited while they were selected for a bariatric surgery. Lean women (3) and men (2) (age 40±12 years, BMI 21±1 kg/m^2,^ fasted glycemia 4.9±1 mM) were recruited while undergoing inguinal hernia, pancreatitis surgery, or lipectomy. The study was performed according to the French Huriet-Serusclat law, with the acceptance of a National Board under the agreement number DGS 2003/0395. Informed written consent was obtained from all subjects. During surgery, surgical subcutaneous adipose tissue biopsies were obtained and immediately frozen in liquid nitrogen and stored at −80°C.

### Cells

3T3-L1 cells were cultured and differentiated into adipocytes as described [22]. To establish 3T3-L1 cell lines stably expressing GFP-Rab4b, 3T3-L1 fibroblasts were infected with the adequate retrovirus, selected in puromycin (2 µg/ml), and differentiated into adipocytes. This procedure allows for low levels of protein overexpression in nearly all the cells. Transfection of siRNA was performed by using INTERFERin™ according to the manufacturer's instructions (Ozyme). Human preadipocytes from lean non diabetic subjects (Biopredic, Rennes, France) were grown, differentiated into adipocytes, and used 10–15 days later as described [42].

### Real-time PCR

RNAs were prepared using the RNeasy total RNA kit (Qiagen, France), treated with DNAse (Ambion Inc), and used to synthetize cDNAs with cDNA High Capacity Archive Kit (Applied Biosystems). Real-time quantitative RT-PCR was performed with the ABI PRISM 7000 sequence Detection System (Applied Biosystems) and SYBR green dye or TaqMan technology [39]. The mRNA levels (R) were expressed relative to levels of the large P0 subunit of the acidic ribosomal phosphoprotein-RPLP0 (named 36B4 in mice) (ΔCt = Ct_R_-Ct_RPLP0_). The relative amount of mRNA between two groups is given by 2^−ΔΔCt^, where ΔΔCt = [ΔCt(R) of obese or diabetic patient]-[mean of ΔCt(R) of lean group]. Statistical significance was determined using the non-parametric Kruskal-Wallis test with the ΔCt of the different groups. p<0.05 was significant.

### Immunofluorescence

Cells on glass coverslips were analyzed by sequential scanning confocal fluorescent microscopy with a PL APO 63×1.4 oil objective (TSP SP, Leica, Deerfield, IL, USA or LSM 510, Zeiss, Göttingen, Germany [22], [40]. The images were combined by using PhotoShop (Mountain View, CA, USA) and analyzed with the ImageJ software. Image acquisition and analyzis were performed on the C3M MicorBio Cell Imaging Facility.

### Quantification of GLUT4 and TfR present at the plasma membrane

3T3-L1 cells were grown on glass coverslips, differentiated into adipocytes, and stimulated or not with insulin for 20 min. Plasma membrane sheets were prepared, immuno-stained with anti GLUT4 or anti TfR antibodies, and quantification of their amount was performed as previously described in [22], [43] using WGA labeling for normalization to the amount of membranes.

### Glucose Transport

3T3-L1 adipocytes were serum deprived for 2 h, treated without or with the indicated concentrations of insulin. Glucose transport was determined by adding 2-[^3^H]deoxyglucose (0.1 mM, 0.5 µCi/ml) for 3 min [22], [42]. Cells were washed four times with ice-cold PBS, lysed and used to assess glucose uptake by scintillation counting. Results were normalized for protein content.

### GLUT4 internalization assay

Adipocytes stably expressing GLUT4myc-DsRed cells were serum deprived before a treatment with 10 nM insulin to trigger GLUT4myc-DsRed at the plasma membrane. Cells were then incubated with anti myc antibodies (25 µg/ml) for 2 h at 4°C in DMEM containing 20 mM Hepes, pH 7.4 and 1% BSA. At the end of this incubation, cells were washed 6 times with ice cold DMEM/Hepes/BSA and then wortmannin (100 nM) was added to inhibit insulin signaling without affecting GLUT4 internalization [44]. Cells were then transferred at 37°C to trigger the internalization of GLUT4, and at different times, paraformaldehyde was added to a concentration of 4%. After 5 min, the paraformaldehyde was quenched with NH_4_Cl. Cells were washed 3 times with PBS and then incubated for 1 h at room temperature with 0.1 µg/ml of HRP-coupled anti mouse antibodies in DMEM/Hepes/BSA. Antibodies only recognized plasma membrane GLUT4. Adipocytes were extensively washed before the quantification of the amount of HRP-coupled anti mouse antibodies by using o-phenylenediamine dihydrochloride as substrate (reference P-9187, Sigma, St Louis, MO, USA). Non specific binding, determined by identically treating adipocytes that did not express GLUT4-myc-DsRed was subtracted from all the values.

### Endosomal ablation through the use of HRP-conjugated transferrin (HRP-Tf)

The endosomal ablation was performed according to Melvin *et al* [45]. 3T3-L1 adipocytes were serum deprived for 2 h before starting an incubation of 2 h with 20 µg/ml of HRP-Tf at 37°C. To eliminate surface-bound HRP-Tf, cells were washed 3 times for 10 min with ice-cold isotonic citrate buffer (150 mM NaCl and 20 mM sodium citrate (pH 5.0). Cells were then washed in ice-cold PBS. Diamino benzidine (DAB) was added (100 µg/ml) to all cells, and H_2_O_2_ was added (0.04%) to one of each pair of wells. After incubation for 1 h at 4°C in the dark, the reaction was stopped by washing in ice-cold PBS containing 5% BSA. Cells were then extensively washed with ice cold PBS, resuspended in Tris pH 7.4 containing 250 mM sucrose and a cocktail of protease inhibitor (completeTM, Roche Diagnostics, Manheim, Germany). After homogenisation by 10 passages through a 22G needle, cells were centrifuged 10 min at 5 000 *g* at 4°C to eliminate non-homogenized cells. Supernatants (total homogenate) were prepared for protein analysis or subjected to a 30 min centrifugation at 19 000 *g* and 4°C to eliminate plasma membranes. Supernatants (intracellular vesicles) were prepared for analysis of the GLUT4, TfR, and tubulin content by Western Blot. Detection of the proteins was made by chemiluminescence with a FUJIFILM LAS-3000 (FUJIFILM France S.A.S, St Quentin en Yvelines, France).

## Supporting Information

Table S1 Sequences of the siRNA used against RAb4a and Rab4b. The sequence of the forward siRNA are given as well as their position relative to the sequence of Rab4a or RAb4b mRNA. All siRNAs targeted the coding sequences thus decreasing the possible side effects linked to miRNA mimicking.
(0.03 MB DOC)

Figure S1 Localization of GFP-Rab4b in a second adipocyte cell line. A. The levels of overexpression of GFP-Rab4b were determined in two independent 3T3-L1 cell lines at the adipocyte stage. The cell line GFP-Rab4b#1 was used in Figures 4–6. The amount of GFP-Rab4b was determined by using an anti GFP. The numbers indicates the quantification of the bands corresponding to GFP-Rab4b normalized by that of tubulin. The amount of Rab4b mRNA (Rab4b+GFP-Rab4b) was determined in each cell line as in the wild type one (wt) by using real time PCR and specific primers designed in the coding sequence. We verified that the adipocytes from each cell lines were identically differentiated by quantifying PPARgamma2, a marker of adipocyte differentiation. B–C. GFP-Rab4b is associated with GLUT4 containing compartment and is partially colocalized with Tfr in GFP-Rab4b#2 adipocytes. Adipocytes were treated like in Figure 4 and 6. Two confocal sections, obtained in the middle and at the bottom of the cells, are shown for GFP-Rab4b (green), GLUT4 (red), and the merge image. Quantifications of the index of colocalization were performed as in Figure 6 and were shown in the panel C. * indicates significant differences with p<0.001 by using the Kruskal-Wallis test. D. Adipocytes were transiently cotransfected with vectors encoding for GFP-Rab4b and myc tagged GLUT4-DsRed and processed for direct fluorescence. E. Adipocytes were transiently cotransfected with vectors encoding for GFP-RAb4a and myc tagged GLUT4-DsRed. Bars are 10 µm.
(2.92 MB TIF)

Figure S2 GFP-Rab4a localization with Glut4. Adipocytes stably expressing GFP-Rab4a were serum deprived overnight before being processed for immonufluorescence. GFP-Rab4a is expressed using anti GFP monoclonal antibody followed like in Figure 4. Two confocal sections of the same cells, obtained in the middle and the bottom of the cells, are shown for GFP-Rab4a (green), GLUT4 (red), and the merge image. Enlarged views of the delineated areas are shown in the right columns. Bar is 1 µm.
(2.17 MB TIF)

Figure S3 GFP-Rab4a localization with TfR. Immunofluorescence of adipocytes expressing GFP-Rab4a were treated as above. GFP-Rab4a was detected using polyclonal anti GFP whereas Tfr was detected with a mAb. Two confocal sections of the same cells, obtained in the middle and the bottom of the cells, are shown for GFP-Rab4a (green), TfR (red), and the merge image. Enlarged views of the delineated areas are shown in the right columns. Bar is 1 µm.
(2.08 MB TIF)

Figure S4 Effect of Rab4a down regulation on glucose uptake and GLUT4 localization in 3T3-L1 adipocytes. A. Adipocytes were transiently transfected with 200 nM of control or anti Rab4a (Sequences provided in supplementary File 2). 72 h later, cells were processed as in Figure 7 in order to measure DOG uptake. B. Cells were treated as in A and GLUT4 localization was determined by indirect immunofluorescence. C. Adipocytes were transiently transfected with 200 nM of control, anti Rab4b, or anti Rab4a siRNA. 72 later the amount of Rab4a and Rab4b mRNA was measured by real time PCR. The results were expressed relative to the amount of each mRNA in control cells. D. Adipocytes were transiently transfected with control, anti Rab4a-1, anti Rab4a-2, or remained untreated (no). 72 h later total homogenates were prepared and the amount of GLUT4, GLUT1, Rab4 and tubulin determined by western blotting with their respective antibodies.
(1.03 MB TIF)

Figure S5 The down regulation of Rab4b did not alter the activity of PKB. Adipocytes were transfected with the indicated siRNA like in Figure 7. 72 h later they were serum deprived and treated with the indicated concentrations of insulin (like in figure 7D). 40 µg of proteins were analyzed for phosphorylated phospho-Thr308 PKB (A) and phosphorylated AS160 (B), as well as total PKB and AS160 for normalization. The results are expressed as the % of the maximal effect in control siRNA-treated adipocytes. The mean +/− SEM of three experiments was shown.
(0.09 MB TIF)
