# Supplementary material for: Rab4b Is a Small GTPase Involved in the Control of the Glucose Transporter GLUT4 Localization in Adipocyte
Source: PLoS One. 2009 Apr 17;4(4):e5257. doi: 10.1371/journal.pone.0005257 (PMC2707114; doi:10.1371/journal.pone.0005257)
Supplement: Table S1 — Sequences of the siRNA used against RAb4a and Rab4b. The sequence of the forward siRNA are given as well as their position relative to the sequence of Rab4a or RAb4b mRNA. All siRNAs targeted the coding sequences thus decreasing the possible side effects linked to miRNA mimicking. (0.03 MB DOC) [file pone.0005257.s001.doc]

| **siRNA** | **Target Sequence** | **Position**  **(CDS :142-783)** | **Supplier** |
| --- | --- | --- | --- |
| Rab4b-1 | GACUGUGAAACUACAGAUU | 306-324 | **Eurogentec** |
| Rab4b-2 | GGACUCCAACCACACUAUC | 246-264 | **Ambion**  Silencer Pre-designed siRNA |
| Rab4b-3 | GAUCCAGGGUGGUCAACGU  CCUAGAGGCUUCUCGCUUU  GGUCGGUGACGCGGAGUUA  CCACCAGUUUAUUGAGAAU | 278-296  540-558  353-371  216-234 | **Dharmacon**  On-TARGETplus SMART pool |

| **siRNA** | **Target Sequence** | **Position**  **(CDS : 141-798)** | **Supplier** |
| --- | --- | --- | --- |
| Rab4a-1 | GCCAGAACAUCGUCCUUAU | 458-476 | **Eurogentec** |
| Rab4a-2 | GGUUCGCACAAGAGAAUGA | 539-557 | **Eurogentec** |
