# Supplementary figures and images for: Rab4b Is a Small GTPase Involved in the Control of the Glucose Transporter GLUT4 Localization in Adipocyte
Source: PLoS One. 2009 Apr 17;4(4):e5257. doi: 10.1371/journal.pone.0005257 (PMC2707114; doi:10.1371/journal.pone.0005257)

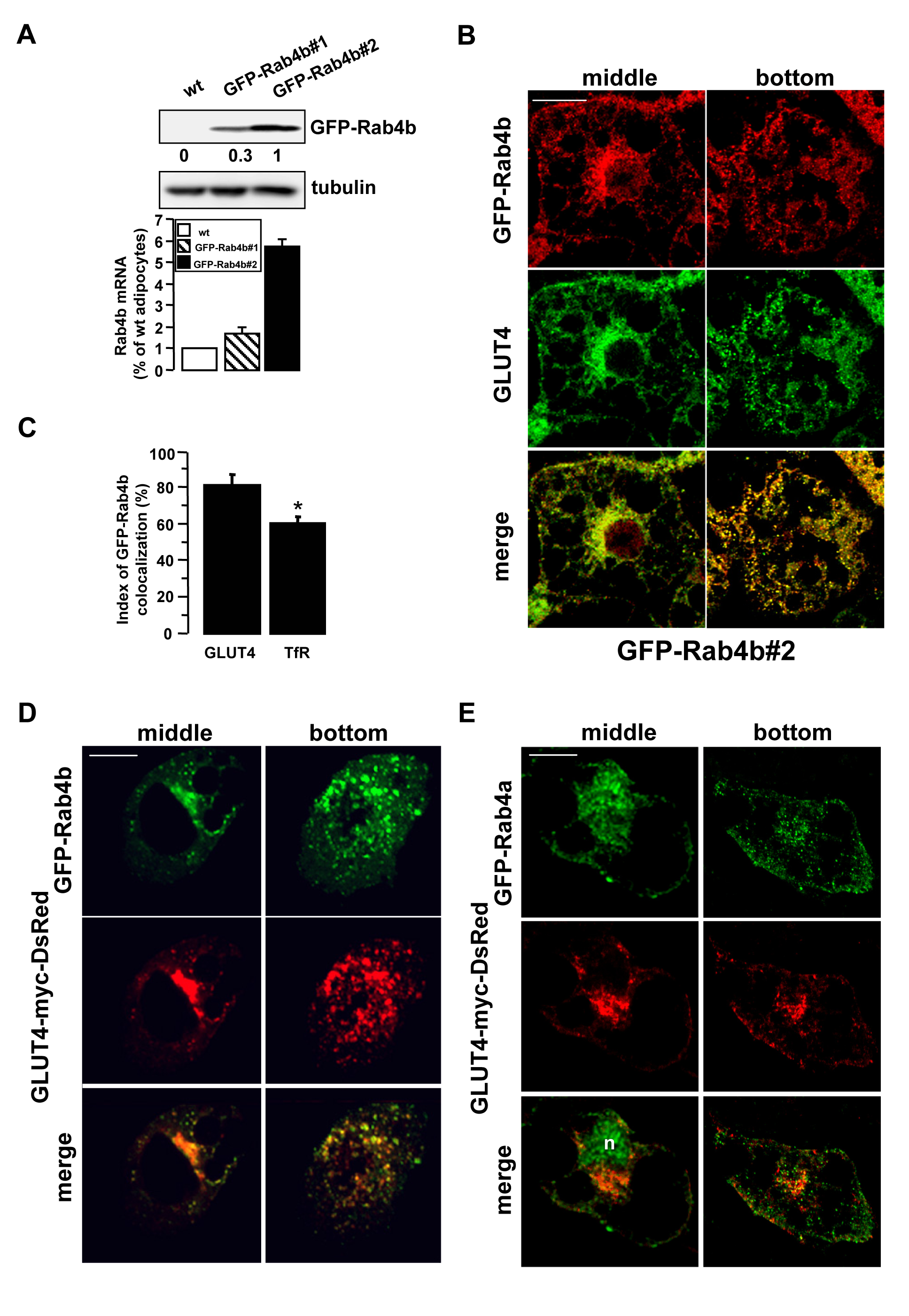

Supplement: Figure S1 — Localization of GFP-Rab4b in a second adipocyte cell line. A. The levels of overexpression of GFP-Rab4b were determined in two independent 3T3-L1 cell lines at the adipocyte stage. The cell line GFP-Rab4b#1 was used in Figures 4– 6. The amount of GFP-Rab4b was determined by using an anti GFP. The numbers indicates the quantification of the bands corresponding to GFP-Rab4b normalized by that of tubulin. The amount of Rab4b mRNA (Rab4b+GFP-Rab4b) was determined in each cell line as in the wild type one (wt) by using real time PCR and specific primers designed in the coding sequence. We verified that the adipocytes from each cell lines were identically differentiated by quantifying PPARgamma2, a marker of adipocyte differentiation. B–C. GFP-Rab4b is associated with GLUT4 containing compartment and is partially colocalized with Tfr in GFP-Rab4b#2 adipocytes. Adipocytes were treated like in Figure 4 and 6. Two confocal sections, obtained in the middle and at the bottom of the cells, are shown for GFP-Rab4b (green), GLUT4 (red), and the merge image. Quantifications of the index of colocalization were performed as in Figure 6 and were shown in the panel C. * indicates significant differences with p<0.001 by using the Kruskal-Wallis test. D. Adipocytes were transiently cotransfected with vectors encoding for GFP-Rab4b and myc tagged GLUT4-DsRed and processed for direct fluorescence. E. Adipocytes were transiently cotransfected with vectors encoding for GFP-RAb4a and myc tagged GLUT4-DsRed. Bars are 10 µm. (2.92 MB TIF) [file pone.0005257.s002.tif]

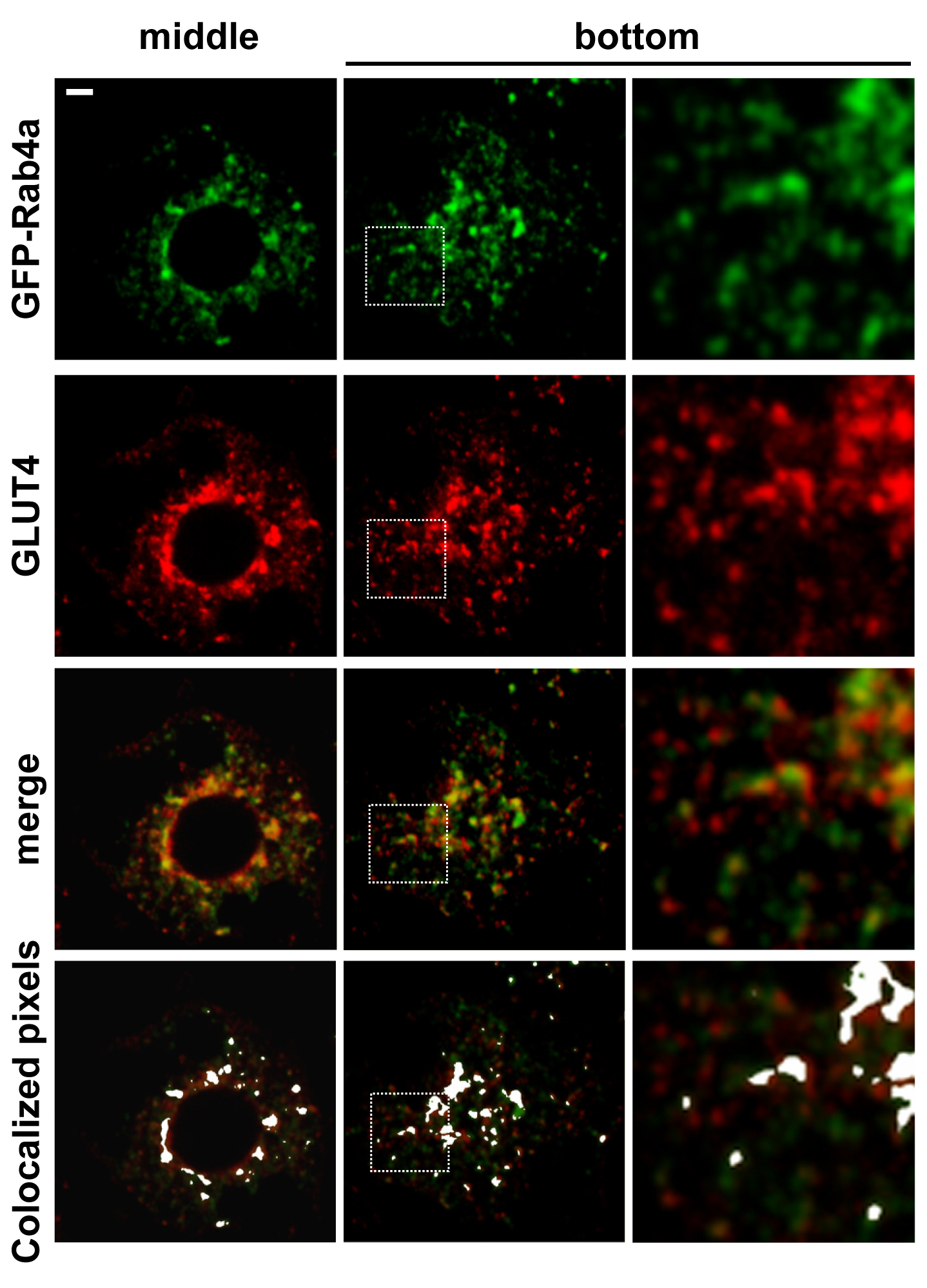

Supplement: Figure S2 — GFP-Rab4a localization with Glut4. Adipocytes stably expressing GFP-Rab4a were serum deprived overnight before being processed for immonufluorescence. GFP-Rab4a is expressed using anti GFP monoclonal antibody followed like in Figure 4. Two confocal sections of the same cells, obtained in the middle and the bottom of the cells, are shown for GFP-Rab4a (green), GLUT4 (red), and the merge image. Enlarged views of the delineated areas are shown in the right columns. Bar is 1 µm. (2.17 MB TIF) [file pone.0005257.s003.tif]

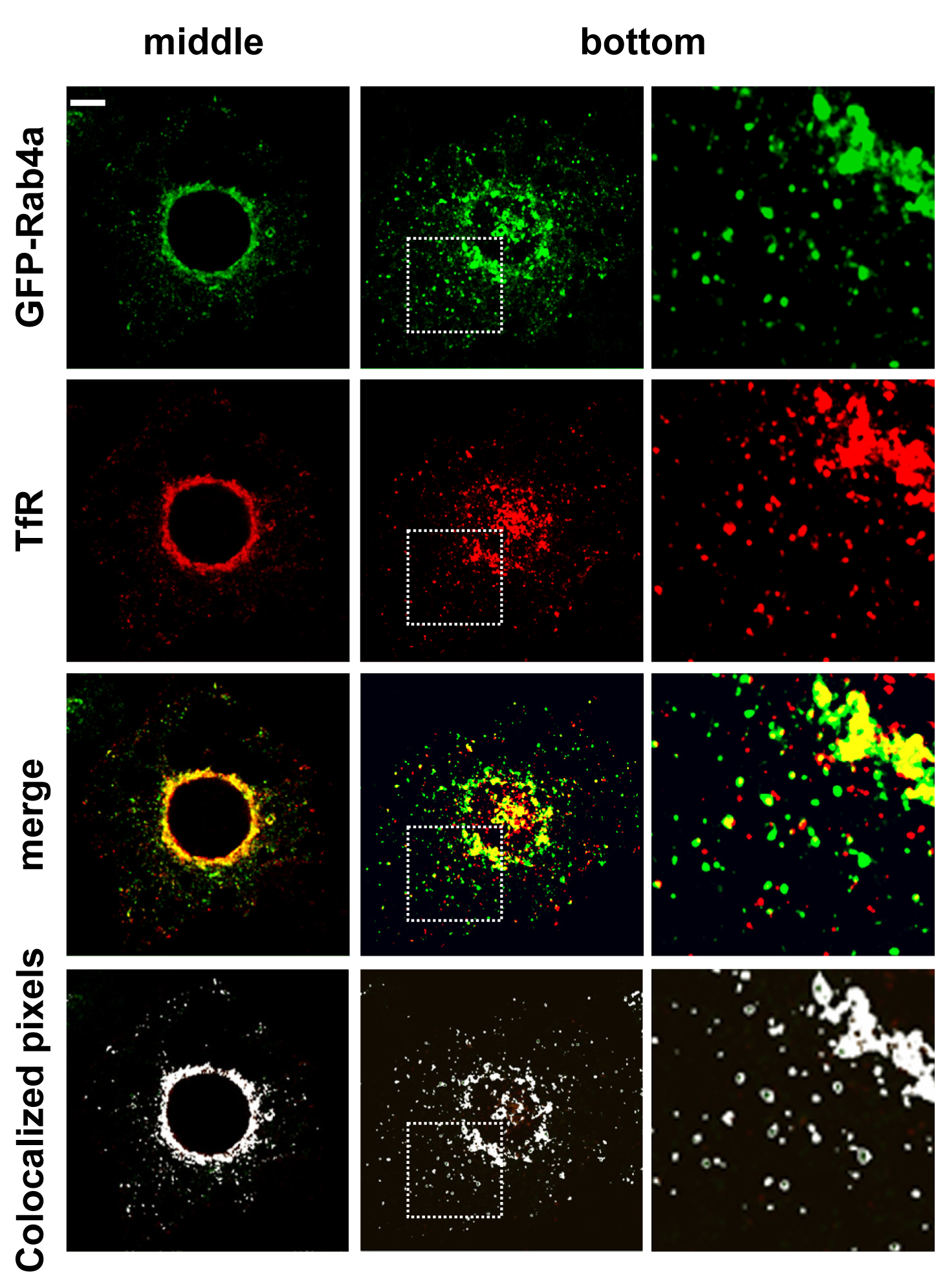

Supplement: Figure S3 — GFP-Rab4a localization with TfR. Immunofluorescence of adipocytes expressing GFP-Rab4a were treated as above. GFP-Rab4a was detected using polyclonal anti GFP whereas Tfr was detected with a mAb. Two confocal sections of the same cells, obtained in the middle and the bottom of the cells, are shown for GFP-Rab4a (green), TfR (red), and the merge image. Enlarged views of the delineated areas are shown in the right columns. Bar is 1 µm. (2.08 MB TIF) [file pone.0005257.s004.tif]

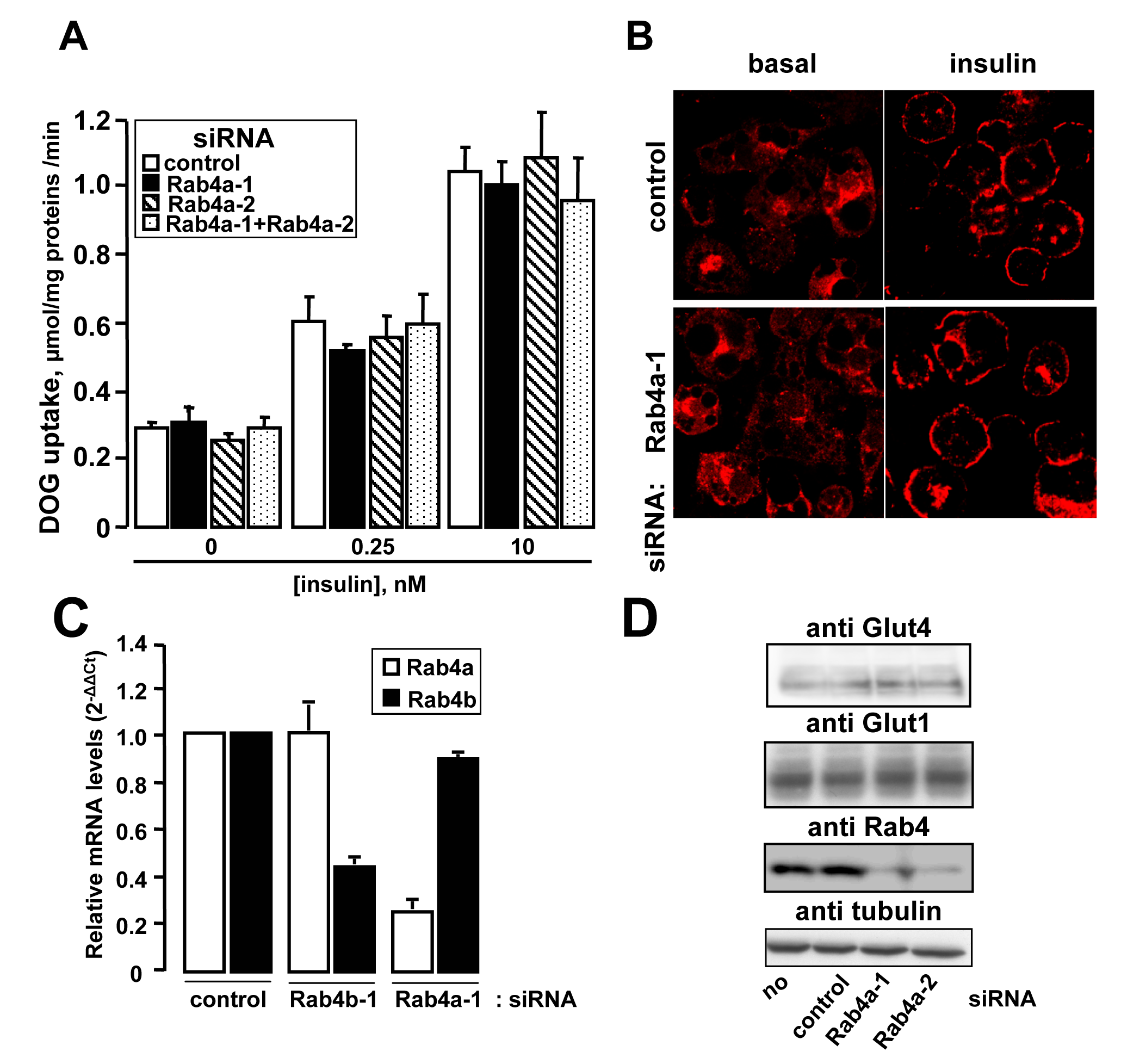

Supplement: Figure S4 — Effect of Rab4a down regulation on glucose uptake and GLUT4 localization in 3T3-L1 adipocytes. A. Adipocytes were transiently transfected with 200 nM of control or anti Rab4a (Sequences provided in supplementary File 2). 72 h later, cells were processed as in Figure 7 in order to measure DOG uptake. B. Cells were treated as in A and GLUT4 localization was determined by indirect immunofluorescence. C. Adipocytes were transiently transfected with 200 nM of control, anti Rab4b, or anti Rab4a siRNA. 72 later the amount of Rab4a and Rab4b mRNA was measured by real time PCR. The results were expressed relative to the amount of each mRNA in control cells. D. Adipocytes were transiently transfected with control, anti Rab4a-1, anti Rab4a-2, or remained untreated (no). 72 h later total homogenates were prepared and the amount of GLUT4, GLUT1, Rab4 and tubulin determined by western blotting with their respective antibodies. (1.03 MB TIF) [file pone.0005257.s005.tif]

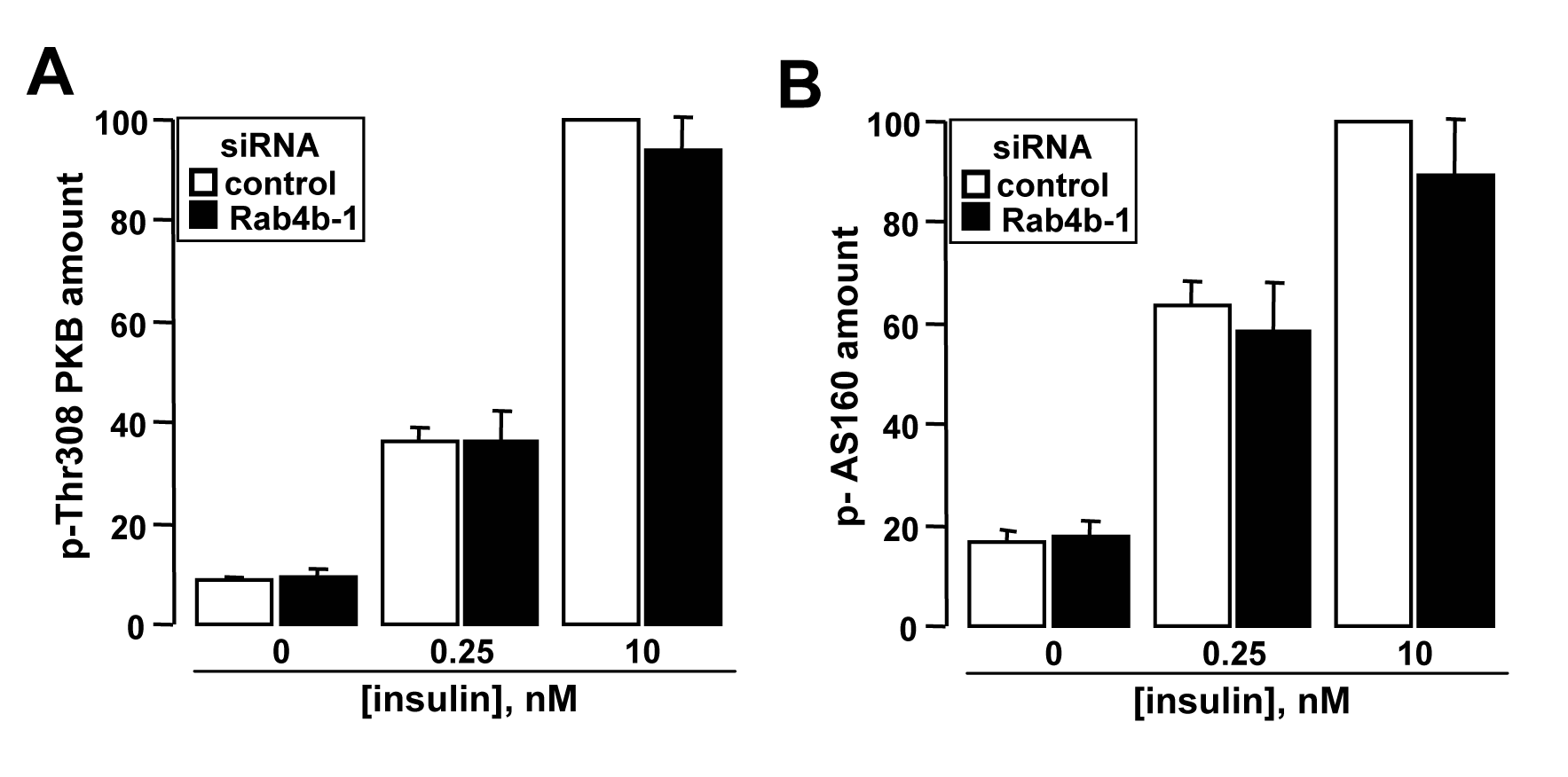

Supplement: Figure S5 — The down regulation of Rab4b did not alter the activity of PKB. Adipocytes were transfected with the indicated siRNA like in Figure 7. 72 h later they were serum deprived and treated with the indicated concentrations of insulin (like in figure 7D). 40 µg of proteins were analyzed for phosphorylated phospho-Thr308 PKB (A) and phosphorylated AS160 (B), as well as total PKB and AS160 for normalization. The results are expressed as the % of the maximal effect in control siRNA-treated adipocytes. The mean +/− SEM of three experiments was shown. (0.09 MB TIF) [file pone.0005257.s006.tif]
